# Exploring the association between *BIN1* gene polymorphisms and hippocampal subfield volume in community mild cognitive impairment

**DOI:** 10.3389/fneur.2025.1525664

**Published:** 2025-02-12

**Authors:** Jiali Luo, Junjiao Ping, Haibo Zhang, Ying Zhang, Zhenkun Tan, Chuijia Kong, Xinxia Liu

**Affiliations:** ^1^Department of Psychiatry, The Third People’s Hospital of Zhongshan City, Zhongshan, China; ^2^Department of Radiology, The Third People’s Hospital of Zhongshan City, Zhongshan, China

**Keywords:** mild cognitive impairment, *BIN1*, rs10200967, hippocampal subfield volumes, IHATA

## Abstract

**Introduction:**

Mild cognitive impairment (MCI) is an early stage of Alzheimer’s disease (AD), crucial for early diagnosis. *BIN1*, a key AD susceptibility gene after *APOE*, has higher brain expression in AD and interacts with tau, affecting its pathology. Specific *BIN1* SNPs are linked to AD and MCI, but mechanisms are unclear. This study will explore how *BIN1* polymorphisms might influence MCI development and correlate with hippocampal integrity in MCI patients using MRI.

**Methods:**

This study enrolled a total of 52 elderly individuals with MCI and 55 cognitively CN individuals from five communities in Zhongshan Torch Development Zone. Blood samples were collected for analysis of *BIN1* rs10200967, rs1060743, and rs4663093 gene polymorphisms, and MRI scans were conducted to assess the volume of hippocampal subregions. The study also seeks to examine the distribution of *BIN1* genotypes in both MCI and healthy control populations, as well as to investigate the potential association between *BIN1* rs10200967, rs1060743, and rs4663093 genotypes and hippocampal subregion structure in individuals with MCI.

**Results:**

Significant structural atrophy was observed in multiple hippocampal subregions, including left cornu ammonis (lCA), left dentate gyrus (lDG), left hippocampal-amygdaloid transition area (lHATA), left subiculum (lSubc), right ornu ammonis (rCA), right dentate gyrus (rDG), right subiculum (rSubc), left entire hippocampus complex (lHIP), and right entire hippocampus complex (rHIP) in seniors with MCI compared to those in the CN (*p* < 0.05), after adjusting for age, gender, education level, and *APOE*ε4 status. Conversely, no significant differences were observed in left entorhinal cortex (lEC), right entorhinal cortex (rEC), right hippocampal-amygdaloid transition area (rHATA), and total intracranial volume (TIV) (*p* > 0.05). Notably, there were no significant differences in the distribution of *BIN1* rs10200967, rs1060743, and rs4663093 genotypes among elderly individuals (*p* > 0.05). Furthermore, the association between the *BIN1* rs10200967 genotype and lHATA atrophy significant in the MCI after adjusting for age, gender, education level, *APOE*ε4 status, and TIV (*p* < 0.05).

**Conclusion:**

This study presents novel findings indicating an association between the *BIN1* rs10200967 genotype and lHATA atrophy, with the rs10200967 CC genotype showing a higher volume of lHATA in individuals with MCI. These results suggest that the rs10200967 CC genotype may confer a protective effect against MCI, offering a potential basis for early detection and prevention of AD.

## Introduction

Alzheimer’s disease (AD) is a progressive neurodegenerative brain disorder characterized by dementia, cognitive impairment, memory loss, severe behavioral abnormalities. The major neuropathological hallmarks of AD include amyloid-beta (Aβ)-containing plaques, neurofibrillary tangles, as well as synapse and neuron loss. To date, no effective treatment for AD has been identified. A cross-sectional study conducted in China revealed an overall dementia prevalence of 6.0%, equating to approximately 15.07 million individuals affected (1). It is noteworthy that AD is currently the fifth leading cause of death among individuals aged 65 years in the United States (2), and brings a heavy burden to society. The incidence rate of AD disease is expected to significantly increase among the elderly as the population ages. Therefore, there is a pressing need for the development of effective therapeutic interventions. Mild cognitive impairment (MCI) typically manifests in the preclinical stage of Alzheimer’s disease, while prodromal AD represents the earliest symptomatic stage with cognitive symptoms that do not meet the criteria for a dementia diagnosis. The conversion rate of MCI to AD is estimated to be approximately 15.4–33.4% per year. Once Alzheimer’s disease is diagnosed, it progresses through an irreversible neurodegenerative process. MCI presents a crucial window of opportunity for potential interventions aimed at prevention. Therefore, it is very important to diagnose MCI as early and accurately as possible.

Recent advancements have revealed additional genes linked to the development of AD. Genome-wide association studies (GWAS) have identified Bridging integrator 1 (*BIN1*) as the second susceptibility gene for AD, following *APOE* (3). *BIN1* is a scaffolding protein that is prominently expressed in the brain and muscle, playing a role in various cellular functions including endocytosis, translational regulation, and signal transduction (4). Human *BIN1* belongs to the amphoteric protein family, which is primarily characterized by its role in endocytosis. Amphoteric proteins facilitate the endocytosis of both neuronal and non-neuronal cells through interactions with various clathrin-coated pit-associated proteins. Furthermore, the dephosphorylation of *BIN1* is essential for the initiation of endocytosis (5). In individuals with AD, there is an upregulation of *BIN1* expression in the brain, and the interaction between the SH3 domain of *BIN1* and the proline-rich domain in tau contributes to the exacerbation of tau pathology (6).

Recent research has highlighted the significance of *BIN1* gene polymorphisms, particularly in relation to AD. Studies have indicated that variations in *BIN1* loci genes, such as rs744373 and rs3747742, may play a role in the pathogenesis of AD. Additionally, limited research suggests a potential link between *BIN1* rs10200967 (7), rs1060743 (8) and rs4663093 (9) abnormalities and the development of MCI and AD. For example, a notable disparity in the prevalence of the *BIN1* rs4663093 variant has been observed between AD patients and healthy individuals, implicating its involvement in the extracellular accumulation of Aβ and hyperphosphorylation of tau protein. Furthermore, MCI patients with Medial temporal lobe atrophy and hippocampal abnormalities have shown similarities to AD patients in this regard, involve Aβ extracellular accumulation and hyperphosphorylation of tau protein (9).

Moreover, when compared to individuals without health issues, those with MCI exhibit a significant reduction in Medial temporal lobe atrophy and hippocampus subfield volumes. The annual rate of hippocampal atrophy and the acceleration of this atrophy may serve as indicators for predicting the progression from MCI to AD (10–12). Several studies have demonstrated that high levels of *BIN1* expression in the hippocampus are associated with tau accumulation and endocytosis in AD (5, 13, 14). Therefore, *BIN1* polymorphisms are implicated in the pathophysiology of both AD and MCI. However, it remains unclear whether there are variations in the distribution of *BIN1* rs10200967, rs1060743 and rs4663093 genotypes in the community in Zhongshan, whether *BIN1* rs10200967, rs1060743 and rs4663093 associated to hippocampus subfield volumes.

Magnetic resonance imaging (MRI) is a robust neuroimaging modality utilized for the assessment of brain structure and function *in vivo*. Through the integration of MRI with neuroimaging biomarkers, the identification of hippocampal abnormalities in patients with MCI is made possible. This methodology aids in the early detection and diagnosis of MCI populations at elevated risk for AD, thereby playing a critical role in the prevention and treatment of AD. In order to improve understanding of the neurological mechanisms involved in disease pathogenesis potentially linked to *BIN1* gene polymorphisms, an analysis was performed on the prevalence of these polymorphisms among individuals with MCI in our region. This study also explored the relationship between *BIN1* gene polymorphisms and the volume characteristics of the hippocampal subfield region of the brain. The objective of this investigation is to lay the groundwork for uncovering the neurodevelopmental pathways associated with MCI.

## Materials and methods

### Subjects

This study was conducted as a component of the “Comprehensive Prevention and Treatment of Key Diseases of the elderly in Zhongshan City” initiative, utilizing a random sampling methodology. Participants were selected from five community centers in the Zhongshan Torch Development Zone between May and October 2023. Of the 497 elderly individuals aged 60 years or older who underwent mammography, 92 were identified as having MCI. Ultimately, the study included a total sample size of 52 individuals with MCI and 55 healthy elderly controls (CN). The subjects with MCI were selected by applying Paterson’s diagnostic criteria of MCI (15, 16): Memory loss within the last year; Age 60 years and above; Right-handed; Normal hearing and eyesight; A Mini Mental Status Examination Scale (MMSE) score ≤ 17 points (illiterate), ≤20 points (education level of primary school), or ≤ 24 points (education level of secondary school or above) respectively; A Activities of daily living (ADL) score ≥ 60 points; physician-confirmed diagnosis of not AD; Signature of informed consent. The inclusion criteria for healthy controls were as follows: Age 60 years and above; Right-handed; Normal hearing and eyesight; MMSE score >17 points (illiterate), >20 points (education level of primary school), or >24 points (education level of secondary school or above) respectively; ADL score ≥ 60 points; physician-confirmed diagnosis of not AD; Signature of informed consent. Exclusion criteria are as follows: a current or history of alcohol/drug abuse; central nervous system trauma; were taking medication which may affect cognitive function; had or previously had any health condition that may influence cognitive function. The study protocol was approved by the Medical Ethics Committee of Zhongshan third people’s hospital (approval number: SSYLL20220401). All participants were informed about the contents of the respective study and gave their written consent to participate.

### Clinical assessment

The study utilized a self-designed questionnaire to gather demographic information from respondents, encompassing variables such as gender, age, education level, marital status, and area of specialization. Cognitive function was assessed using the MMSE (17), which evaluates various cognitive domains including orientation, registration, attention, calculation, language, and recall through a total of 30 items scored on a binary scale. ADL were evaluated to assess subjects’ ability to perform daily tasks, with a scale consisting of 10 questions related to daily activities (18), the higher the score the worse the self-care ability.

### Biospecimen collection

All participants fasted overnight (not less than 8 h), and blood was collected in EDTA-treated 5 mL vacutainer tubes and gently inverted and mixed. After collection, the specimens were placed in universal transport boxes, stored at 4°C, and transported to laboratory within 4 h of collection. Plasma and blood cell pellets were separated from EDTA-treated blood samples by centrifugation at 3000 rpm for 10 min at 4°C, stored at −80°C for later processing.

### Gene polymorphism test

The TIANamp Genomic DNA kit was used to extract blood cell pellets sample DNA. The DNA concentration was detected by an ultra-micro spectrophotometer and confirmed DNA integrity through agarose gel electrophoresis.

The MassARRAY SNP genotyping platform (Agena Bioscience, San Diego, CA, United States) was employed for genotyping SNPs within the *BIN1* and *APOE* genes. Primers were designed utilizing the Agena primer design tool^1^. The polymerase chain reaction (PCR) primers were diluted to a concentration of 100 μM, and the PCR primer mixture was prepared in accordance with the 1EXT 200 protocol. The extension primer mixture was formulated using a 1:25 dilution of the primers. Following the finalization of the extension primer mixture configuration, 2 μL of each primer were further diluted at a 1:25 ratio for subsequent mass spectrometric analysis. The proportion of extension primers for each individual locus was adjusted based on the results of the tests. In preparation for PCR amplification, a PCR master mix was formulated in a 1.5-ml Eppendorf tube, achieving a total volume of 4.1 μL. This mix comprised 1.850 μL of water, 0.625 μL of PCR buffer containing 15 mM MgCl2, 0.325 μL of additional MgCl2, 0.100 μL of deoxyribonucleoside triphosphate (dNTP) mix, 0.1000 μL of primer mix, and 0.200 μL of HotStarTaq polymerase (Qiagen, Hilden, Germany). Utilizing an 8- or 12-channel pipette, 4 μL of the master mix was dispensed into each well of a 384-well plate, followed by the addition of 1 μL of genomic DNA at a concentration of 20 ng/μl. The samples were subsequently mixed and subjected to centrifugation at 1,000 rpm for 1 min. To mitigate evaporation during the PCR process, the 384-well plates were sealed with a protective film. The PCR amplification protocol was executed as follows: an initial denaturation at 94°C for 5 min; 45 cycles consisting of denaturation at 94°C for 20 s, annealing at 56°C for 30 s, and extension at 72°C for 1 min; a final extension at 72°C for 3 min; and a hold at 4°C. The PCR products were subjected to treatment with shrimp alkaline phosphatase (SAP) to eliminate free dNTPs from the reaction mixture. A reaction mixture consisting of 1.53 μL of water, 0.17 μL of SAP buffer, and 0.3 μL of SAP was prepared in a 1.5-ml Eppendorf tube. Subsequently, 2 μL of this reaction mixture was added to each well of a 384-well PCR plate containing 5 μL of the PCR product. The SAP reaction was conducted in a PCR machine under the following conditions: 37°C for 20 min, followed by 85°C for 5 min, and a final hold at 4°C. Following the alkaline phosphatase treatment, a single-base extension reaction was performed. After the PCR process, Na+, Mg2+, K+, and other salt ions were removed using cation exchange resin to minimize the occurrence of excessive peaks in the mass spectrometry analysis spectrum, which could compromise the accuracy of the results. A microvolume of the purified sample was subsequently loaded onto the SpectroCHIP using a MassARRAY nanodispenser to facilitate the preparation of a co-crystalline film of the chip matrix and the sample.

### Structural MRI preprocessing

MRI plays a crucial role in both research on MCI and in clinical studies, particularly in the assessment of significant hippocampal abnormalities through structural MRI (19). The MRI data utilized in this study were obtained using a 3.0 Tesla Vida Siemens MRI Scanner (Siemens, Erlangen, Germany) equipped with a 64-channel head coil. To minimize head movement and scanner noise, foam pads and headphones were employed during data collection. High-resolution structural images were acquired using a three-dimensional magnetization-prepared rapid gradient-echo (3D MPRAGE) T1-weighted sequence. The scanning parameters included a repetition time (TR) = 2000 ms, an echo time (TE) = 2.26 ms, an inversion time (Tl) =900 ms, a specific slice thickness = 1 mm, interslice gap = 0 mm, Voxel size = 1 × 1 × 1 mm3, number of slices =176, flip angle (FA) = 8°, field of view (FOV) =256mmx256mm, matrix =256×256 and length of the time course = 4.06 min. Structural MRI data were analyzed using voxel-based morphometry, with each scan being visually inspected by experienced neuroradiologists to identify and exclude any artifacts or gross anatomical abnormalities, The neuroradiologists were blinded to genetic information. Structural image preprocessing was conducted using the SPM12^2^ and CAT12^3^ toolbox in MATLAB (version R2016b, The MathWorks, Inc., Natick, MA, USA), which involved segmenting into gray matter, white matter, and cerebrospinal fluid. Subsequently, the images were non-linearly normalized to MNI space using the DARTEL algorithm. Finally, the segmented GM images were smoothed with an 8 mm full-width-half-maximum (FWHM) isotropic Gaussian kernel. To mitigate boundary effects between gray matter and white matter, an absolute threshold masking of 0.1 was implemented in this study. In this study, we will concentrate on the hippocampus and its subregions as subcortical regions of interest within the brain. Drawing upon previous research (20), and acknowledging the inherent complexity, we utilized the shape and anatomical variation of the hippocampal region, along with the probability map of hippocampal cellular structure, to delineate it into the following five subregions: Cornu Ammonis (CA), Dentate Gyrus (DG), Entorhinal Cortex (EC), Subiculum (Subc), and the hippocampal-amygdaloid transition area (HATA). To mitigate partial volume effects, the subfields were aggregated as follows: CA1, CA2, and CA3 were combined into CA; CA4 and DG were merged into DG (21). Subsequently, a total of 10 hippocampal subregion masks for both the left and right hemispheres were generated, and the gray matter volumes of these 10 regions were extracted for intergroup comparison. Eventually, 41 individuals with MCI and 45 healthy controls underwent MRI scans for the study.

### Statistical analysis

The data entry was conducted using Epidata3.1, while statistical analysis was performed using SPSS 26.0. The Hardy–Weinberg genetic equilibrium test was applied to the subjects’ genotypes using the *χ*2 test for goodness of fit. Data that exhibited normal distribution were presented as mean ± standard deviation and analyzed using ANCOVA. Conversely, data that did not adhere to normal distribution were presented as median (upper quartile, lower quartile) and analyzed using nonparametric statistical methods. Categorical data were analyzed using the chi-squared test. The comparison of hippocampal subfield volumes between individuals with MCI and control groups was conducted through analysis of covariance, with adjustments made for age, gender, education level, and *APOE*ε4 as covariates. Additionally, the examination of hippocampal subfield volumes differences among individuals with MCI with varying *BIN1* genotypes was carried out using a general linear model, with adjustments made for age, gender, education level, *APOE*ε4, and total intracranial volume (TIV) as covariates. A two-tailed test was used, and a difference of *p* < 0.05 was considered statistically significant.

## Results

### Sample characteristics

A total of 107 community-dwelling older adult participants aged 60 and above, diagnosed with MCI (*n* = 52) and CN (*n* = 55), were included in the study. There were no significant differences observed between the MCI and CN groups in terms of mean age (72.98 ± 7.38 vs. 73.35 ± 6.85, *p* > 0.05), gender distribution (male: female,9/43 vs. 17/38, *p* > 0.05), educational levels (12/18/25 vs. 17/15/20, *p* > 0.05), and *APOE*ε4 (1/51 vs. 3/52, *p* > 0.05). The mean MMSE score was significantly higher in the MCI group compared to the CN group (9.00 vs. 5.00, *p* < 0.05), as depicted in Table 1.

**Table 1:** Demographic characteristics of both groups (*n* = 107).

		MCI (*n* = 52)	CN (*n* = 55)	*χ*^2^/*Z*/*t*	*p-*value
Gender (m/f)		9/43	17/38	2.688	0.101
Age		72.98 ± 7.38	73.35 ± 6.85	0.045	0.791
Education	Illiterate	12	17	1.608	0.448
	Primary	18	15		
	Secondary school and above	25	20		
*APOE*ε4 (−/+)		1/51	3/52	0.926	0.336
MMSE		18.00 (14.00, 22.75)	27.00 (24.00, 29.00)	−7.500	0.001

### Test of Hardy–Weinberg equilibrium and locus polymorphism analysis

Genotype data for the rs10200967, rs1060743 and rs4663093 polymorphisms in the *BIN1* gene for MCI (*n* = 52) and CN (*n* = 55) were tested for Hardy–Weinberg Equilibrium. There were no significant differences between the observed genotype frequencies and allele distribution at these loci, as shown in Table 2. The distribution of genotype frequencies at each locus showed that the genotype at the *BIN1* locusrs10200967 was predominantly CT in both MCI and CN, with 51.9% in MCI and 45.4% in CN. The rs1060743 genotype was predominantly AG, with 48.1% in MCI and 49.1% in CN. The rs4663093 genotype was predominantly CC, with 69.2% in MCI and 72.7% in CN.

**Table 2:** Genotype and allele frequencies of SNPs within *BIN1* among the MCI and CN.

SNP	Group (*n*)	*PHWE*	Alleles (%)		*χ*2	*p*	Genotypes (%)			*χ*2	*p*
			C	T			CC	CT	TT		
rs10200967	MCI (*n* = 52)	1	53 (51.0)	51 (49.0)	0.142	0.706	13 (25.0)	27 (51.9)	12 (23.1)	0.800	0.670
	CN (*n* = 55)	0.588	61 (55.4)	49 (44.5)			18 (32.7)	25 (45.4)	12 (21.8)		
			A	G			AA	AG	GG		
rs1060743	MCI (*n* = 52)	0.786	55 (53.0)	49 (47.0)	0.915	0.339	15 (28.8)	25 (48.1)	12 (23.1)	1.140	0.565
	CN (*n* = 55)	0.781	63 (57.3)	43 (42.7)			18 (32.7)	27 (49.1)	8 (14.5)		
			C	T			CC	CT	TT		
rs4663093	MCI (*n* = 52)	1	87 (83.6)	17 (16.4)	0.133	0.715	36 (69.2)	15 (28.8)	1 (2.0)	0.161	0.923
	CN (*n* = 55)	1	94 (85.4)	16 (24.6)			40 (72.7)	14 (25.4)	1 (1.9)		

### Comparison of the hippocampal subfield volumes between MCI and CN

**Figure 1:**
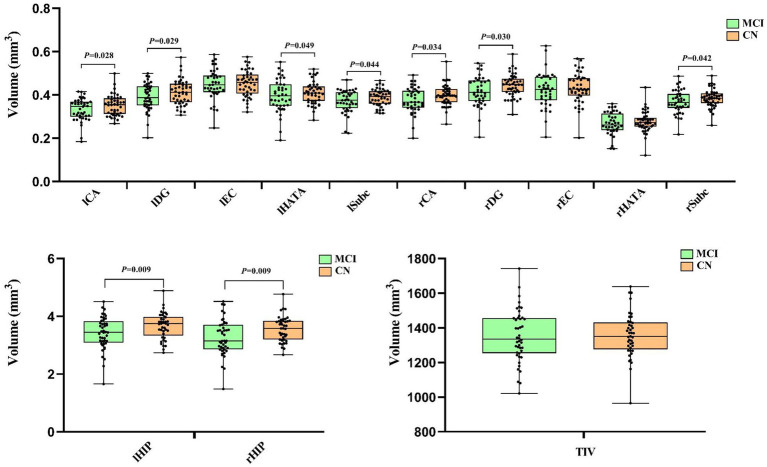
The comparison of Hippocampus subfield volumes between the MCI and CN. Left cornu ammonis (lCA), left dentate gyrus (lDG), left entorhinal cortex (lEC), left hippocampal-amygdaloid transition area (lHATA), left subiculum (lSubc), left entire hippocampus complex (lHIP), right ornu ammonis (rCA), right dentate gyrus (rDG), right entorhinal cortex (rEC), right hippocampal-amygdaloid transition area (rHATA), right subiculum (rSubc), right entire hippocampus complex (rHIP) and total intracranial volume (TIV). The comparison of hippocampus subfield volumes between MCI and CN after adjusting for age, gender, education level and Aopeε4 status. Data in this figure analyzed with ANCOVA.

In the 41 MCI and 45 CN participants included in the hippocampal subfield volumes MRI analysis, the Comparisons of the hippocampal subfield volumes were performed using a general linear model procedure, adjusting for age, gender, education level, and *APOE*ε4 status as covariates. The findings revealed that the lCA, lDG, lHATA, lSubc, rCA, rDG, rSubc, lHIP and rHIP were also significantly decreased in the MCI group compared to the CN group (both *p* < 0.05), while the lEC, rEC, rHATA and TIV were non-significant decreased in the two groups (*p* > 0.05), as shown in Figure 1.

### Comparison of hippocampal subfield volumes in different genotypes of MCI *BIN1* genotype

A covariate ANOVA was done with age, gender, education level, *APOE*ε4 status and TIV to compare the structural differences in the hippocampal subfield volumes between different genotypes of *BIN1* rs10200967, rs1060743 and rs4663093 in the MCI group. The results showed statistically significant differences in IHATA among different genotypes (*p* < 0.05), and there were no significant differences in lCA, lDG, lEC, lSubc, rCA, rDG, rEC, rHATA, rSubc, lHIP and rHIP according *BIN1* rs10200967 genotypes (*p* > 0.05). We did not find any significant results with respect to differences in lCA, lDG, lEC, lHATA, lSubc, rCA, rDG, rEC, rHATA, rSubc, lHIP and rHIP among the *BIN1* rs1060743 and rs4663093 genotypes (*p* > 0.05), As shown in Table 3.

**Table 3:** The comparison of hippocampus subfield volumes with different genotypes of rs10200967, rs1060743 and rs4663093.

SNP	Group	lCA	lDG	lEC	lHATA	lSubc	rCA	rDG	rEC	rHATA	rSubc	lHIP	rHIP
rs10200967	CC (*n* = 9)	0.37 ± 0.01	0.43 ± 0.02	0.45 ± 0.02	0.38 ± 0.02	0.39 ± 0.01	0.40 ± 0.01	0.43 ± 0.02	0.44 ± 0.02	0.30 ± 0.01	0.39 ± 0.01	2.03 ± 0.07	1.97 ± 0.07
	TT (*n* = 10)	0.36 ± 0.01	0.42 ± 0.02	0.45 ± 0.02	0.35 ± 0.01	0.38 ± 0.01	0.38 ± 0.01	0.41 ± 0.02	0.45 ± 0.02	0.28 ± 0.01	0.38 ± 0.01	1.96 ± 0.07	1.90 ± 0.06
	CT (*n* = 22)	0.35 ± 0.01	0.40 ± 0.02	0.42 ± 0.01	0.33 ± 0.01	0.36 ± 0.01	0.37 ± 0.01	0.40 ± 0.01	0.41 ± 0.01	0.27 ± 0.01	0.36 ± 0.01	1.86 ± 0.05	1.81 ± 0.04
	*F*	1.425	1.228	1.159	3.941	1.509	1.199	1.134	1.961	2.189	2.528	2.049	2.106
	*p*-value	0.255	0.306	0.326	**0.029**	0.236	0.314	0.334	0.157	0.128	0.095	0.145	0.138
rs1060743	AA (*n* = 14)	0.36 ± 0.01	0.42 ± 0.01	0.45 ± 0.02	0.35 ± 0.01	0.38 ± 0.01	0.38 ± 0.01	0.42 ± 0.01	0.44 ± 0.02	0.28 ± 0.01	0.38 ± 0.01	1.95 ± 0.06	1.90 ± 0.06
	GG (*n* = 7)	0.38 ± 0.02	0.43 ± 0.02	0.43 ± 0.02	0.35 ± 0.01	0.38 ± 0.01	0.39 ± 0.02	0.43 ± 0.02	0.42 ± 0.03	0.27 ± 0.02	0.37 ± 0.01	1.97 ± 0.09	1.88 ± 0.09
	AG (*n* = 20)	0.35 ± 0.01	0.43 ± 0.01	0.43 ± 0.01	0.34 ± 0.01	0.37 ± 0.01	0.37 ± 0.01	0.40 ± 0.01	0.42 ± 0.01	0.28 ± 0.01	0.37 ± 0.01	1.89 ± 0.05	1.85 ± 0.05
	*F*	1.149	0.833	0.319	0.099	0.317	0.376	0.640	0.381	0.050	0.646	0.443	0.265
	*p*-value	0.329	0.444	0.729	0.906	0.731	0.690	0.534	0.686	0.951	0.79	0.646	0.769
rs4663093	CC + TT (*n* = 27)	0.35 ± 0.01	0.41 ± 0.01	0.43 ± 0.01	0.34 ± 0.01	0.37 ± 0.01	0.38 ± 0.01	0.41 ± 0.01	0.42 ± 0.01	0.28 ± 0.01	0.37 ± 0.01	1.90 ± 0.04	1.87 ± 0.04
	CT (*n* = 14)	0.37 ± 0.01	0.42 ± 0.01	0.45 ± 0.01	0.35 ± 0.01	0.38 ± 0.01	0.38 ± 0.01	0.41 ± 0.01	0.44 ± 0.02	0.27 ± 0.01	0.37 ± 0.01	2.00 ± 0.04	1.88 ± 0.06
	*F*	1.043	0.847	1.896	0.043	0.329	0.000	0.021	0.778	0.334	0.105	0.831	0.018
	*p*-value	0.314	0.364	0.177	0.837	0.570	0.991	0.885	0.384	0.567	0.748	0.369	0.894

Left cornu ammonis (lCA), left dentate gyrus (lDG), left entorhinal cortex (lEC), left hippocampal-amygdaloid transition area (lHATA), left subiculum (lSubc) left entire hippocampus complex (lHIP), right ornu ammonis (rCA), right dentate gyrus (rDG), right entorhinal cortex (rEC), right hippocampal-amygdaloid transition area (rHATA), right subiculum (rSubc) and right entire hippocampus complex (rHIP). The comparison of hippocampus subfield volumes with different genotypes of rs10200967, rs1060743 and rs4663093 after adjusting for age, gender, education level, Aopeε4 status and total intracranial volume (TIV). Data in this table are expressed as mean ± SD and analyzed with ANCOVA. Bold represents *P* < 0.05.

## Discussion

As a new susceptibility gene for AD, *BIN1* is located on chromosome 2q14.3. Previous research has suggested that polymorphisms in the *BIN1* gene may be linked to an increased risk of developing AD potentially through its role in lipid droplet endocytic transport (22). Abnormal endocytosis in AD patients leads to decreased transport and clearance of brain Aβ, contributing to the onset of the disease. *BIN1* is the second most important genetic risk factor in AD. *BIN1* risk-allele carriers show accelerated tau-PET accumulation at higher Aβ levels (23). SNP rs744373 of *BIN1* increased the risk of developing AD in populations from East China (24). Other studies could not confirm the association between *BIN1* rs744373 risk-allele and elevated [^18^F]AV1451 signal in CN older adults or MCI (25). Additionally, polymorphisms at the rs7561528 and rs6733839 loci of the *BIN1* gene, as well as the rs1057233 locus of the SPI1 gene, have been suggested to be associated with MCI in the Chinese Han population (22). In this study, SNPs linked to AD within the *BIN1* gene were selected for analysis, and their correlation with hippocampal changes in individuals with MCI was examined. In this study, we found no significant differences in the frequency and distribution of *BIN1* SNP rs10200967, rs1060743, and rs4663093 between the MCI group and the healthy control group. This phenomenon may be attributed to the fact that the samples were derived from individuals with MCI, whose disease progression has not yet advanced to the level observed in AD patients. Furthermore, the development of MCI may be influenced by the interaction of multiple genes rather than the presence of a single gene polymorphism exerting a predominant effect.

Prior research has indicated that hippocampal atrophy is a risk factor for the progression from MCI to dementia and is considered a precursor to AD (26). In our study, after adjusting for age, gender, education level, and *APOE*ε4 status, we observed structural differences in various hippocampal subregions, including lCA, lDG, lHATA, lSubc, rCA, rDG, rSubc, lHIP, and rHIP, between individuals with MCI and CN. These findings support the presence of a trajectory of hippocampal subregion atrophy during the MCI stage, which aligns partially with previous research (27, 28). This study supports the utility of MRI technology in assessing hippocampal subregions in elderly individuals as a means of early intervention and prevention of AD, potentially surpassing the efficacy of clinical and behavioral assessments. Nevertheless, our findings revealed no statistically significant distinctions in the volumes of the lEC, rEC, rHATA and TIV between the two groups, suggesting that hippocampal subregion atrophy varies in degree during the development of MCI, specific hippocampal subregions may offer superior predictive value for the onset of AD compared to the entire hippocampus (29).

**Figure 2:**
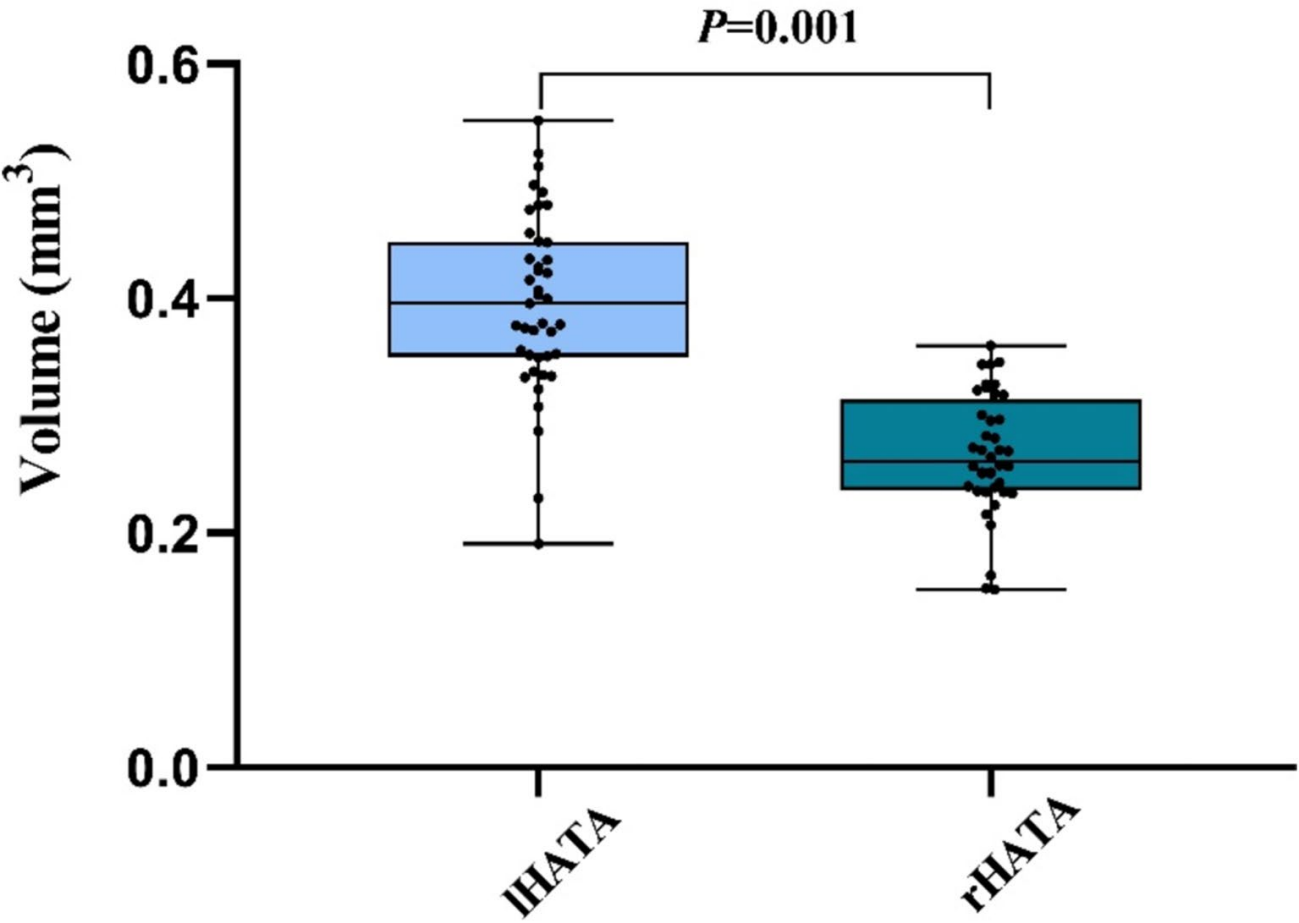
The comparison between lHATA and rHATA in MCI. Left hippocampal-amygdaloid transition area (lHATA) and right hippocampal-amygdaloid transition area (rHATA). Data in this figure analyzed with *T*-test.

Our observation revealed a significant difference in the volume of the lHATA to the rHATA, suggesting a higher susceptibility to neurodegenerative changes in the left hemisphere (Figure 2). Tsai’s et al. study on an AD mouse model also found an increase in *β*-amyloid deposition in the left hemisphere, resulting in greater atrophy in the left hippocampal subregions compared to the right (30). Previous research indicates that aging and neurodegenerative disorders frequently demonstrate hemispheric differences in atrophy patterns, with studies showing uneven hippocampal atrophy between individuals with mild cognitive impairment and cognitively normal individuals. In conclusion, our study found that the left hemisphere is more susceptible to degeneration compared to the right hemisphere (30, 31), resulting in greater atrophy in the lHATA compared to the rHATA.

In this study, after adjusting for age, gender, education level, *APOE*ε4 status and TIV, we investigated the relationship between *BIN1* SNP rs10200967, rs1060743 and rs4663093 genotypes and the volume of hippocampal subregions in individuals with MCI. Our analysis included a comparison of hippocampal subregion structural differences among different *BIN1* genotypes. The findings revealed a significant association between the lHATA volume in MCI individuals with SNP rs10200967, with the CC genotype showing the largest volume, followed by TT and CT having the smallest volume. These findings indicate that there are notable differences in HATA among individuals with different *BIN1* genotypes, particularly highlighting the CT genotype of rs10200967 as a potentially protective factor against MCI. HATA, situated in the medial portion of the hippocampus, is considered to play a crucial role in information processing within the hippocampal-amygdala network. The degeneration of HATA may compromise the structural integrity of this network, which is essential for effective information processing (32, 33). The variations in atrophy volume observed may be linked to cognitive and memory decline associated with normal aging (34) and Parkinson’s disease (33). This indicates that the *BIN1* rs10200967 variant may play a role in the pathophysiological mechanisms underlying information processing, cognitive function, and memory in individuals with MCI. These results underscore the genetic influence of various *BIN1* genotypes on the morphology of the hippocampal region and their significance in the likelihood of developing AD.

However, it is important to acknowledge the limitations of this study, including the relatively small sample size and the reliance on cross-sectional data without considering the longitudinal relationship between *BIN1* gene polymorphism, MCI, and hippocampal subregion atrophy. Furthermore, our study focused solely on alterations in hippocampal subregions through the utilization of structural MRI data. Additionally, the correlation analysis between *BIN1* SNP rs10200967 and lHATA did not incorporate clinical symptoms in the analysis. Thus, it is imperative for future investigations to confirm these results with a larger sample size. Moreover, integrating multimodal imaging techniques like functional MRI and electroencephalography, and examining the relationship between *BIN1* gene polymorphism, clinical symptoms, and hippocampal regions longitudinally, would offer a more holistic comprehension of MCI pathology.

This research examined the volumes of hippocampal subregions in individuals with MCI and healthy control subjects, and established that atrophy in these subregions may act as a predisposing factor for the advancement of MCI to dementia, manifesting prior to the identification of clinical symptoms. In the elderly MCI, no differences were observed in the distribution of SNP rs10200967, rs1060743, and rs4663093. Among these, individuals with the CC genotype of *BIN1* rs10200967 had larger hippocampal subregion volumes in lHATA, followed by those with TT and CT genotypes, suggesting a protective role of the *BIN1* rs10200967 CC genotype in MCI, potentially linked to the lHATA subregion of the hippocampus. These results not only validate but also build upon prior research on *BIN1* gene polymorphisms and hippocampal subregions in the context of MCI, offering additional insights into hippocampal atrophy for the early identification of dementia.

## Data Availability

The raw data supporting the conclusions of this article will be made available by the authors, without undue reservation.

## Footnotes

^1^ http://agenacx.com/

^2^https://www.fil.ion.ucl.ac.uk/spm, version 7,771

^3^ https://neuro-jena.github.io/cat12-help/
